# Symptom profiles and inflammatory markers in moderate to severe COPD

**DOI:** 10.1186/s12890-016-0330-1

**Published:** 2016-12-03

**Authors:** Huong Q. Nguyen, Jerald R. Herting, Kenneth C. Pike, Sina A. Gharib, Gustavo Matute-Bello, Soo Borson, Ruth Kohen, Sandra G. Adams, Vincent S. Fan

**Affiliations:** 1 Kaiser Permanente Southern California, 100 S. Los Robles, Pasadena, CA 91101 USA; 2 University of Washington, Seattle, USA; 3 University of Washington & Puget Sound Veterans Administration, Seattle, USA; 4 University of Texas Health Science Center at San Antonio and The South Texas Veterans Health Care System, San Antonio, Texas USA

**Keywords:** COPD, Symptoms, Dyspnea, Fatigue, Pain, Depression, Anxiety, Inflammation

## Abstract

**Background:**

Physical and psychological symptoms are the hallmark of patients’ subjective perception of their illness. The purpose of this analysis was to determine if patients with COPD have distinctive symptom profiles and to examine the association of symptom profiles with systemic biomarkers of inflammation.

**Methods:**

We conducted latent class analyses of three physical (dyspnea, fatigue, and pain) and two psychological symptoms (depression and anxiety) in 302 patients with moderate to severe COPD using baseline data from a longitudinal observational study of depression in COPD. Systemic inflammatory markers included IL1, IL8, IL10, IL12, IL13, INF, GM-CSF, TNF-α (levels >75^th^centile was considered high); and CRP (levels >3 mg/L was considered high). Multinominal logistic regression models were used to examine the association between symptom classes and inflammation while adjusting for key socio-demographic and disease characteristics.

**Results:**

We found that a 4-class model best fit the data: 1) low physical and psychological symptoms (26%, Low-Phys/Low-Psych), 2) low physical but moderate psychological symptoms (18%, Low-Phys/Mod Psych), 3) high physical but moderate psychological symptoms (25%, High-Phys/Mod Psych), and 4) high physical and psychological symptoms (30%, High-Phys/High Psych). Unadjusted analyses showed associations between symptom class with high levels of IL7, IL-8 (*p* ≤ .10) and CRP (*p* < .01). In the adjusted model, those with a high CRP level were less likely to be in the High-Phys/Mod-Psych class compared to the Low-Phys/Low-Psych (OR: 0.41, 95%CI 0.19, 0.90) and Low-Phys/Mod-Psych classes (OR: 0.35, 95%CI 0.16, 0.78); elevated CRP was associated with in increased odds of being in the High-Phys/High-Psych compared to the High-Phys/Mod-Psych class (OR: 2.22, 95%CI 1.08, 4.58). Younger age, having at least a college education, oxygen use and depression history were more prominent predictors of membership in the higher symptom classes.

**Conclusions:**

Patients with COPD can be classified into four distinct symptom classes based on five commonly co-occurring physical and psychological symptoms. Systemic biomarkers of inflammation were not associated with symptom class. Additional work to test the reliability of these symptom classes, their biological drivers and their validity for prognostication and tailoring therapy in larger and more diverse samples is needed.

**Trial registration:**

Clinicaltrials.gov, NCT01074515.

## Background

Symptoms are the hallmark of patients’ experience of illness and their responses to it, and patients with chronic obstructive pulmonary disease (COPD) experience a high burden of symptoms such as dyspnea, fatigue, anxiety and depression. COPD is a heterogeneous disease [1, 2] with patients experiencing different patterns of common symptoms [3, 4]. In a recent study, Park and Larson [3] found that COPD patients tended to cluster in either two or three groups based on the overall burden of dyspnea, fatigue, depression and anxiety, and that symptom clusters had a stronger association with mortality than individual symptoms. Recent evidence also suggests that pain is an additional symptom that contributes to the overall symptom burden in COPD that has not previously been well-recognized [5].

Understanding the biological mechanisms that underlie the inter-individual variability in patients’ symptom experiences could help inform future treatment approaches. Previous research suggests that differences in symptom experiences may be due to an individual’s ability to respond to physical and psychological stressors through changes in pro- and anti-inflammatory cytokines. Administration of inflammatory agents has been shown to induce “sickness behaviors” with subjects reporting fatigue, depression, anxiety, sleepiness, and hyperalgesia [6, 7] The majority of studies to date in COPD have focused on the association between depressive symptoms and selected pro-inflammatory markers with some studies showing a positive relationship [8, 9], whereas others did not [10–13] Very little is known regarding the relationship between markers of systemic inflammation and common co-occurring COPD symptoms such as dyspnea, fatigue, pain, depression, and anxiety.

Therefore, the first aim of this paper were to determine if patients with stable COPD can be classified into distinct symptom classes based on the severity of their physical (dyspnea, fatigue and pain) and psychological symptoms (depression and anxiety). The second aim was to determine the association between these symptom classes and systemic biomarkers of inflammation.

## Methods

### Study design/settings

The COPD Activity: Serotonin Transporter, Cytokines and Depression (CASCADE) cohort is a multi-site prospective observational study of COPD patients who were followed for 2 years to study the biological causes and functional consequences of depression. This manuscript is a cross-sectional descriptive analysis of data from 302 patients collected at entry to CASCADE. This study was approved by the respective institutional review boards at three clinical sites: University of Washington, Seattle (37332), VA Puget Sound Health Care System (00240), and University of Texas Health Science Center at San Antonio/South Texas Veterans Health Care System (HSC20100373H) and was registered with ClinicalTrials.gov (NCT01074515).

### Participants

We recruited patients from queries of medical records and pulmonary function tests, chest clinics from the three medical centers, a research database maintained by the investigators, pulmonary rehabilitation programs, better breathers groups, community pulmonary practices, advertisements, study web site, and other referrals. The inclusion criteria were: 1) Clinical diagnosis of COPD 2) Post-bronchodilator forced expiratory volume in one second to forced vital capacity ratio (FEV_1_/FVC) < 70%; 3) Moderate to very severe disease with an FEV_1_ < 80%; 4) Age > 40 years; and 5) Current or past cigarette smoking (>10 pack-years); 6) Stable disease with no acute exacerbations of COPD in the past 4 weeks; 7) Ability to speak, read and write English. Because this study was focused on COPD-related inflammation we excluded patients who reported any of the following conditions: other chronic lung diseases (e.g. asthma, bronchiectasis, cystic fibrosis, or idiopathic pulmonary fibrosis), uncompensated heart failure (exacerbation in the past 4 weeks), primary pulmonary vascular disease, chronic antibiotic use or ongoing infection, autoimmune disease, lung cancer or metastatic cancer, chronic renal failure requiring dialysis, chronic uncompensated liver disease, HIV/AIDS, or chronic oral prednisone use. As this study was focused on depression, we also excluded those with bipolar disease, psychotic disorders, and dementia.

### Procedures

Informed consent was obtained prior to clinic assessments, which included pre- and post-bronchodilator spirometry performed according to American Thoracic Society standards using an EasyOne spirometer (ndd Medical Technologies Inc, Andover, MA) and completion of questionnaires and a six minute walk test. Two days after this clinic visit, a depression and anxiety assessment was completed via telephone by a trained mental health professional.

### Measures

*Demographic* data included self-reported age, gender, education, income, living situation, marital status, and smoking status.

*Disease severity* included distance covered on a six minute walk test, body mass index, self-report of chronic conditions (Charlson co-morbidity index), oxygen supplementation, and post bronchodilator FEV1.

*History of Depression* was measured using the Structured Clinical Interview for Depression (SCID) [14] Any score other than zero on the SCID (indicating at least one episode of depression during a participant’s lifetime, excluding the current episode of depression if any) was considered to be positive. The age of when the first episode occurred was also captured.

*Dyspnea* was measured with the Shortness of Breath Questionnaire (SOBQ) [15] *Fatigue* was measured with the Chronic Respiratory Questionnaire (CRQ-Fatigue subscale) [16] *Pain* was measured with the pain subscale of the Medical Outcomes Study Short-Form 36 [17] *Depression and Anxiety* were measured with the Hospital Anxiety and Depression Scale [18].

*Markers of Systemic Inflammation* included high sensitivity C-reactive protein (CRP), and a panel of inflammatory cytokines: Interleukin (IL)-1, IL-2, IL-4, IL-5, IL-6, IL-7, IL-8, IL-10, IL12, IL13, Interferon (INF), Granulocyte macrophage-colony stimulating factor (GM-CSF), and Tumor necrosis factor (TNF-α). These markers were selected because they were either related to COPD severity or depression based on the published literature [19].

Peripheral blood was collected by venipuncture into vacutainer tubes with ethylenediaminetetraacetic acid anticoagulant. Blood was collected between 9:30 AM and 4:00 PM at each in-clinic assessment. Plasma was obtained by centrifugation of tubes at 2000 X g for 10 min. The samples were stored at -70 0C until analyzed. The concentrations of CRP were measured using a duoset ELISA (R&D Systems); the lower limit of detection was 15.5 pg/mL. The remaining cytokines were measured using the Luminex multiplex platform with Millipore Milliplex High Sensitivity Human Cytokine Magnetic Beads. The lower limit of detection was 0.13 pg/mL. A cut off of >3 mg/L was used to classify patients as having high levels of CRP; for the remaining inflammatory markers, values greater than the 75^th^ percentile was considered high levels of inflammation.

### Data analysis

We conducted latent class/profile analyses [20] of three physical symptoms (dyspnea, fatigue, and pain) and two psychological symptoms (depression and anxiety) to identify distinct classes (subgroups) of symptom profiles. Patients were assigned a probability of being in each of the identified classes with the goal of creating a model that uniquely assigned a subject to a given class (e.g. Pr(ClassA) = 1.0; Pr(ClassB) = 0.0), or at minimum, provided a distinctively high probability to a given class versus all others (e.g. .95 versus .05). Model fit was evaluated using information criteria fit indices (Bayesian Information Criterion, BIC and Akaike’s Information Criterion, AIC); low values indicate model parsimony. We also used other criteria to identify a meaningful fit of model and data and these included class interpretability (the extent to which additional classes provided unique information), class prevalence (preferring classes with at least 2% of the sample for improved replicability), and entropy (a measure of classification based on posterior probability values, with higher values representing better classification). We used analysis of variance and Chi square tests to examine unadjusted differences in socio-demographic characteristics, disease severity, and inflammatory markers across the four classes. Covariates from unadjusted models were included in the final adjusted multinomial logistic regression model if they contributed substantially to model fit, were considered clinically important, or in the case of inflammatory markers, those with a *p* value <0.10. We used MPlus (version 5.0, Los Angeles, CA) for the latent profile analyses and STATA (version 14, College Station, TX) for the multinomial logistic regressions. A *p* value <0.05 was considered statistically significant.

## Results

### Symptom classes

**Fig. 1:**
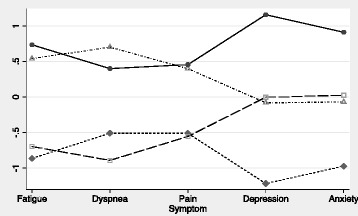
Pattern of COPD symptom classes based on normalized symptom scores. *Z-scores on Y-axis; higher z-scores indicate worse symptoms. Class 1 (*diamond*): Low physical and low psychological symptom burden (Low-Phys/Low-Psych); 26%. Class 2 (*square*): Low physical and moderate psychological symptom burden (Low-Phys/Mod-Psych); 18%. Class 3 (*triangle*): High physical and moderate psychological symptom burden (High-Phys/Mod-Psych); 25%. Class 4 (*circle*): High physical and high psychological symptom burden (High-Phys/High-Psych); 30%

Two-, three-, four-, and five-class solutions were tested as possible fits to the data. Overall, the statistical criteria for model fit (Bayesian and Akaike’s) and classification (entropy) combined with clinical relevance suggested that the four-class solution provided the best representation of the data (BIC = 6026, AIC = 6002, and entropy = 0.87). In the four-class solution, the first class included patients with the lowest burden of physical and psychological symptoms (26%, “Low-Phys/Low-Psych”), the second included patients with low physical but moderate psychological symptoms (18%, “Low-Phys/Mod-Psych”), the third included patients with high physical and moderate psychological symptoms (25%, “High-Phys/Mod-Psych”), and the fourth included patients with the worst physical and psychological symptoms (30%, “High-Phys/High-Psych”). The mean raw values and standardized z-scores for dyspnea, fatigue, pain, anxiety and depression across the four symptom classes are shown in Table 1 and Fig. 1, respectively.

**Table 1:** Socio-demographic characteristics and disease severity across four symptom classes

		Class 1	Class 2	Class 3	Class 4	*P* Value
	Total Sample	Low-Phys/Low-Psych	Low-Phys/Mod-Psych	High-Phys/Mod-Psych	High-Phys/High-Psych	
Variables	*n* = 302	(*n* = 80)	(*n* = 55)	(*n* = 77)	(*n* = 90)	
Socio-Demographics						
Age, years	68 ± 9	72 ± 9	68 ± 8	68 ± 8	64 ± 8	<.001^‡†§^°^∞^
Females	59 (20%)	8 (10%)	10 (18%)	16 (21%)	25 (28%)	.04^§^
Education, some college+	234 (77%)	54 (68%)	48 (87%)	62 (81%)	70 (78%)	.05
Income, > $20,000/year	184 (62%)	22 (29%)	16 (29%)	34 (44%)	43 (48%)	.02
Live with others	223 (74%)	61 (76%)	39 (71%)	57 (74%)	66 (73%)	.92
Currently smoking	86 (28%)	17 (21%)	19 (35%)	24 (31%)	26 (29%)	.35
Disease Severity/Burden						
6-min walk test (feet)	1085 ± 371	1136 ± 370	1196 ± 310	984 ± 344	1061 ± 407	.005^∫^
FEV1% predicted	45.0 ± 15.8	48.1 ± 18.0	45.3 ± 14.6	42.0 ± 13.3	44.4 ± 16.2	.12
O2 supplementation	100 (33%)	18 (23%)	11 (20%)	33 (43%)	38 (42%)	.002^†§∫^°
Number of co-morbidities						.39
0	139 (46%)	37 (46%)	27 (49%)	30 (39%)	45 (50%)	
1	96 (32%)	30 (38%)	14 (25%)	25 (32%)	27 (30%)	
2 or more	67 (22%)	13 (16%)	14 (25%)	22 (29%)	18 (20%)	
Body mass index (BMI)	28.2 ± 6.1	27.8 ± 4.8	27.3 ± 5.1	29.5 ± 6.5	27.9 ± 7.1	.15
Physical Symptoms						
Dyspnea, SOBQ (↓0–120)	42.4 ± 22.7	30.8 ± 18.2	22.1 ± 11.7	58.4 ± 16.8	51.5 ± 21.3	<.001^‡†§∫^°
Fatigue, CRQ (4–28↑)	15.8 ± 4.8	20.0 ± 3.6	19.2 ± 2.7	13.2 ± 3.1	12.3 ± 3.7	<.001^†§∫^°
Pain, SF-36 (0–100↑)	61.8 ± 24.3	74.2 ± 22.1	75.3 ± 22.7	52.1 ± 19.4	50.8 ± 21.7	<.001^†§∫^°
Psychological Symptoms						
Anxiety, HAD-A (↓0–21)	5.0 ± 3.9	1.5 ± 1.3	4.7 ± 2.5	4.3 ± 2.6	8.9 ± 3.6	<.001^‡†§^°^∞^
Depression, HAD-D (↓0–21)	4.2 ± 4.1	0.3 ± 0.5	3.2 ± 2.0	2.9 ± 1.7	9.4 ± 2.8	<.001^‡†§^°^∞^
Depression history						<.001^†§^
None	101 (33%)	45 (56%)	19 (35%)	23 (30%)	14 (16%)	
First episode < = 40 y/o	137 (45%)	23 (29%)	25 (45%)	35 (45%)	54 (60%)	
First episode > 40 y/o	64 (21%)	12 (15%)	11 (20%)	19 (25%)	22 (24%)	

Values are presented as mean ± SD or n(%); Phys = Physical Symptoms; Psych = Psychological Symptoms; Mod: Moderate. SOBQ = Shortness of Breath Questionnaire; CRQ = Chronic Respiratory Questionnaire; SF-36 = Short Form Health Survey Pain Scale; HAD-D = Hospital Anxiety & Depression Scale; HAD-A = Hospital Anxiety & Depression Scale; score ranges with arrows indicating direction of better scores. Post-hoc pairwise contrasts, *p* < .05: ^‡^Class1 vs. Class2, ^†^Class 1 vs. Class 3, ^§^Class 1 vs. Class 4, ^∫^Class 2 vs. Class 3, °Class 2 vs. Class 4, ^∞^Class 3 vs. Class 4

### Unadjusted associations between symptom class membership and inflammatory markers

Unadjusted differences in socio-demographic characteristics, disease severity, and inflammatory markers across the four symptom classes are shown in Tables 1 and 2. The higher symptom classes tended to have younger patients, a greater proportion of women and those with lower income (all, *p* < 0.05) In terms of disease severity, the six minute walk test distance was lower and oxygen use was more common in the higher symptom classes whereas there was no difference in FEV1 % predicted, BMI or number of comorbidities across the four symptom classes. Patients who had their first depression episode before 40 years of age were more likely to be in the highest symptom class. As for the inflammatory markers, 18–36% of patients had cytokine levels greater than the 75^th^ percentile across symptom classes and for CRP, a higher percentage (31–60%) had levels greater than 3 mg/L. Only CRP (*p* = .005) was different across groups, followed marginally by IL-7 (*p* = .08) or IL-8 (*p* = .10).

**Table 2:** Inflammatory markers across four symptom classes

	Mean (95% CI) for Total Sample	Class 1 Low-Phys/Low-Psych	Class 2 Low-Phys/Mod-Psych	Class 3 High-Phys/Mod-Psych	Class 4 High-Phys/High-Psych	*P* Value
Variables	*n* = 302	(*n* = 80)	(*n* = 55)	(*n* = 77)	(*n* = 90)	
Interleukin-1 (IL-1), pg/mL	0.6 (0.2,1.5)	18 (23%)	16 (29%)	19 (25%)	25 (28%)	0.80
Interleukin-2 (IL-2), pg/mL	2.7 (1.0,7.1)	18 (23%)	18 (33%)	20 (26%)	22 (24%)	0.59
Interleukin-4 (IL-4), pg/mL	17.3 (7.1, 42.3)	22 (28%)	15 (27%)	16 (21%)	25 (28%)	0.71
Interleukin-5 (IL-5), pg/mL	0.9 (0.4,1.9)	20 (25%)	13 (24%)	18 (23%)	26 (29%)	0.84
Interleukin-6 (IL-6), pg/mL	2.9 (1.8, 5.0)	22 (28%)	16 (29%)	23 (30%)	16 (18%)	0.25
Interleukin-7 (IL-7), pg/mL	2.4 (1.1, 4.7)	29 (36%)	12 (22%)	17 (22%)	19 (21%)	0.08
Interleuken-8 (IL-8), pg/mL	3.7 (2.7, 5.7)	27 (34%)	17 (31%)	17 (22%)	17 (19%)	0.10
Interleukin-10 (IL-10), pg/mL	18.3 (10.5, 37.4)	24 (30%)	14 (25%)	18 (23%)	21 (23%)	0.74
Interleukin-12 (IL-12), pg/mL	3.9 (1.8, 8.4)	22 (28%)	17 (31%)	15 (19%)	23 (26%)	0.48
Interleukin-13 (IL-13), pg/mL	0.5 (0.1, 3.9)	20 (25%)	18 (33%)	20 (26%)	20 (22%)	0.57
Interferon (IFN), pg/mL	9.2 (4.1, 21.0)	19 (24%)	17 (31%)	16 (21%)	25 (28%)	0.55
Granulocyte macrophage-colony stimulating factor (GM-CSF), pg/mL	2.8 (1.3, 6.9)	20 (25%)	17 (31%)	17 (22%)	23 (26%)	0.72
Tumor necrosis factor (TNF-α), pg/mL	4.7 (3.3, 6.8)	25 (31%)	14 (25%)	21 (27%)	17 (19%)	0.31
High sensitivity C-reactive protein (CRP), mg/L	3.2 (1.5, 6.0)	42 (52%)	33 (60%)	23 (31%)	43 (48%)	0.005^†∫∞^

Values for the total sample are presented as median (IQR); by symptom class, n (%) > 75^th^ percentile or % of patients with CRP > 3 mg/L. Phys = Physical Symptoms; Psych = Psychological Symptoms; Mod: Moderate. Post-hoc pairwise contrasts, *p* < .05: ^†^Class 1 vs. Class 3, ^∫^Class 2 vs. Class 3, ^∞^Class 3 vs. Class 4

### Adjusted associations between symptom class membership and inflammatory markers

Results of the multinomial adjusted logistic regression analyses are shown in Table 3. Only CRP was significantly associated with symptom class, where patients with a high CRP level were less likely to be in the High-Phys/Mod-Psych class compared to both the Low-Phys/Low-Psych (OR: 0.41, 95%CI 0.19, 0.90) and Low-Phys/Mod-Psych classes (OR: 0.35, 95%CI 0.16, 0.78); interestingly, elevated CRP was associated with in increased odds of being in the High-Phys/High-Psych symptom class compared to the High-Phys/Mod-Psych class (OR: 2.22, 95% CI 1.08, 4.58).

**Table 3:** Multinomial logistic regression model predicting symptom class membership

Variables	Low-Phys/Mod-Psych vs. Low-Phys/Low-Psych	High-Phys/Mod-Psych vs. Low-Phys/Low-Psych	High-Phys/High-Psych vs. Low-Phys/Low-Psych	High-Phys/Mod-Psych vs. Low-Phys/Mod-Psych	High-Phys/High-Psych vs. Low-Phys/Mod-Psych	High-Phys/High-Psych vs. High-Phys/Mod-Psych
	Odds Ratio (95%CI)	Odds Ratio (95%CI)	Odds Ratio (95%CI)	Odds Ratio (95%CI)	Odds Ratio (95%CI)	Odds Ratio (95%CI)
Socio-Demographics						
Age, years	0.94* (0.89–0.99)	0.94* (0.89–0.99)	0.87*** (0.83–0.92)	1.00 (0.94–1.05)	0.93** (0.88–0.98)	0.93** (0.89–0.98)
Females	1.73 (0.56–5.33)	1.89 (0.65–5.50)	1.99 (0.70–5.66)	1.09 (0.42–2.85)	1.15 (0.46–2.89)	1.06 (0.48–2.32)
Education, some college+	4.33** (1.57–11.9)	3.63** (1.46–9.00)	2.71* (1.10–6.69)	0.84 (0.29–2.44)	0.63 (0.22–1.79)	0.75 (0.32–1.74)
Income, > $20,000/year	0.96 (0.41–2.25)	0.65 (0.29–1.46)	0.63 (0.28–1.41)	0.68 (0.30–1.53)	0.66 (0.30–1.46)	0.97 (0.49–1.94)
Currently smoking	1.31 (0.50–3.44)	1.37 (0.53–3.56)	0.89 (0.35–2.27)	1.05 (0.43–2.58)	0.68 (0.28–1.63)	0.65 (0.29–1.43)
Disease Severity/Burden						
6-min walk test (for every 100 feet change)	1.00 (0.88–1.14)	0.91 (0.81–1.03)	0.91 (0.80–1.02)	0.91 (0.81–1.03)	0.91 (0.81–1.02)	0.99 (0.90–1.10)
FEV1% predicted	0.99 (0.96–1.02)	0.99 (0.96–1.01)	1.01 (0.98–1.04)	1.00 (0.97–1.03)	1.02 (1.00–1.05)	1.03*(1.00–1.05)
O2 supplementation	0.93 (0.33–2.63	2.02 (0.81–5.05)	3.18*(1.21–8.36)	2.16 (0.84–5.59)	3.41* (1.29–9.01)	1.58 (0.71–3.51)
Number of co-morbidities						
0	Reference	Reference	Reference	Reference	Reference	Reference
1	0.84 (0.35–2.05)	1.42 (0.60–3.33)	1.16 (0.49–2.71)	1.68 (0.68–4.16)	1.37 (0.56–1.33)	0.82 (0.38–1.78)
2 or more	2.14 (0.75–6.14)	2.76 (0.97–7.85)	1.91 (0.65–5.62)	1.29 (0.48–3.44)	0.89 (0.33–2.40)	0.69 (0.28–1.69)
Body mass index	1.01 (0.93–1.09)	1.05 (0.98–1.13)	1.01 (0.94–1.08)	1.04 (0.97–1.12)	1.00 (0.93–1.07)	0.96 (0.91–1.02)
Depression history						
None	Reference	Reference	Reference	Reference	Reference	Reference
First episode < = 40 y/o	1.70 (0.70–4.12)	2.66* (1.10–6.41)	6.31*** (2.49–16.0)	1.56 (0.63–3.90)	3.71** (1.41–9.71)	2.37 (0.99–5.69)
First episode > 40 y/o	1.61 (0.57–4.59)	3.11*(1.15–8.45)	6.55*** (2.27–18.9)	1.93 (0.68–5.49)	4.07* (1.36–12.2)	2.10 (0.79–5.59)
IL-7 pg/ml > 75^th^ centile	0.61 (0.23–1.59)	0.62 (0.24–1.55)	0.81 (0.33–2.04)	1.01 (0.37–2.78)	1.33 (0.50–3.58)	1.32 (0.55–3.17)
IL-8 pg/ml > 75^th^ centile	0.81 (0.33–1.96)	0.42 (0.17–1.04)	0.36* (0.14–0.89)	0.52 (0.20–1.32)	0.44 (0.18–1.10)	0.85 (0.35–2.04)
hsCRP > 3 mg/dl	1.18 (0.53–2.59)	0.41*(0.19–0.90)	0.92 (0.42–1.99)	0.35*(0.16–0.78)	0.78 (0.36–1.70)	2.22*(1.08–4.58)

*Phys* physical, *Psych* psychological, *Mod* moderate

*p < .05; **p < .01; ***p < .001

We found that several socio-demographic and disease severity characteristics were associated with symptom class. In general, older age was associated with a lower risk of being in the higher symptom classes compared to younger age (OR: 0.87 to 0.94). Having a college education was associated with a two to four fold odds of being in a higher symptom class compared to those with lower symptom levels (OR: 2.71 to 4.33). Oxygen use was associated with higher odds of membership in the High-Phys/High-Psych compared to both the Low-Phys/Low-Psych (OR: 3.18, 95%CI 1.21, 8.36) and the Low-Phys/Mod-Psych (OR: 3.41, 95%CI 1.29, 9.01) symptom classes. Patients with a depression history had two to six times the odds of being in the High-Phys/Mod-Psych or High-Phys/High-Psych symptom classes compared to being in the Low-Phys/Low-Psych or Low-Phys/Mod-Psych classes (OR: 2.66 to 6.55). Gender, living situation, smoking status, six minute walk test performance, FEV1% predicted, BMI, or number of co-morbidities did not distinguish across the four symptom classes.

## Discussion

We identified four distinct symptom classes based on three common physical (dyspnea, fatigue, and pain) and two psychological (depression and anxiety) symptoms in a sample of patients with COPD: 1) low physical and psychological symptoms (26%), 2) low physical but moderate psychological symptoms (18%), 3) high physical but moderate psychological symptoms (25%), and 4) high physical and psychological symptoms (30%). Systemic inflammation as measured by 14 serum biomarkers did not appear to have any consistent relationship with these empirically identified symptom classes with the exception of CRP. Another notable finding was that younger age and depression history were associated with higher odds of membership in the higher symptom classes.

Our study differs from two previous reports using data from ~600 participants randomized to the control group of the National Emphysema Treatment Trial (NETT). Those studies used a hierarchical clustering approach and identified two or three symptom subgroups compared to the four classes we identified [3, 4]. Patients enrolled in NETT all had very severe COPD with air-trapping and few comorbidities, and therefore differ from our study sample. Another difference was that we used pain as a fifth measure of symptom burden, and although pain is not traditionally thought of as a COPD-related symptom, a recent large observational study found that pain and dyspnea are often inter-related [5]. In addition, although we found that patients tended to have either high or low physical symptom burden, psychological burden tended to be grouped into low (essentially no psychological symptoms), moderate (some psychological symptoms) and high (psychological symptoms that are in the range of clinically meaningful depressive and anxiety symptoms). The observation of a distinct class of patients who have high physical but only moderate levels of psychological symptoms challenge the common notion that physical and psychological symptoms are tightly coupled in patients with COPD [21] Confirmation of such a symptom cluster in other COPD cohorts is needed.

Contrary to our hypothesis, we did not find that measures of systemic inflammation were associated with membership in symptom classes with the exception of CRP. The relationships between CRP and symptom class were however, not always consistent. Elevated CRP levels were associated with a lower odds of belonging to the High-Phys/Mod-Psych class compared to the Low-Phys/Low-Psych or Low-Phys/Mod-Psych class yet the odds of being in the High-Phys/High-Psych class compared to the High-Phys/Mod-Psych class were greater for those with higher CRP. Although COPD is associated with increased systemic inflammation compared to not having COPD, there is evidence that patients with severe COPD and frequent exacerbations experience immune down-regulation due to chronic stimulation of the immune system [22, 23]. This could explain the paradoxical effects of lower CRP levels in patients with *only high physical symptoms and moderate psychological symptoms.* In contrast, the mechanisms underlying the elevated CRP levels for patients with both high physical and psychological symptoms may be quite different. It is possible that in these patients, high psychological symptom burden precedes and exaggerates the perception of physical symptoms partly through a somatization pathway that is not tightly linked to the severity of COPD per se [24].

The finding from the ECLIPSE study that not all patients with COPD have elevated systemic markers of inflammation partly corroborates our null findings between symptom severity and nearly all the measured biomarkers; in fact, only 16% of the ECLIPSE cohort of over two thousand patients had persistent inflammation and one third never showed evidence of systemic inflammation as measured with 34 biomarkers after a year of follow up [25]. Several early studies in both COPD and other clinical populations reported that higher levels of inflammatory markers were associated with worse fatigue and depression [9, 26–28] In contrast, more recent studies found no relationship between systemic inflammation and depression with authors concluding that patients’ symptom experience is likely driven more by other factors, e.g. perception of disability and underlying psychiatric history, rather than physiological changes [10–13]. It is possible that the underlying biological relationship between physical and psychological symptoms and circulating cytokines is far more complex than we were able to appreciate with our analyses and that the significant associations with only CRP could be spurious.

Our finding showing that younger age and depression history but not gender were predictive of membership in the High-Phys/High-Psych class deserves further comment. It is important to note that our assessment of depression history only includes previous episodes, not any current depression. A significantly higher number of patients in this class experienced their first depression episode during the first three decades of life compared to the other classes. Thus, younger age may be a reflection of an early and persistent psychiatric history in this subgroup of patients who experience more severe physical symptoms despite similar levels of airflow obstruction. Moreover, previous studies show that patients with a lifetime history of depression are twice as likely to smoke compared to someone without such a history [29] and have greater difficulty with smoking cessation, not engage in self-care, [30] and thus, experience a worse disease trajectory. Interestingly, we did not find a difference in smoking exposure, at least based on the self-reported number of years patients smoked in this sample. In contrast to previous studies, [31, 32] we did not find that women were more likely to experience worse physical and psychological symptoms compared to men. The absence of a gender effect on symptom class may be due to insufficient power with a smaller number of women (20%) in this sample. However, it is plausible that after simultaneously adjusting for other relevant variables as we had done, especially depression history, that women with COPD are no more likely to report worse physical and psychological symptoms compared to men.

### Limitations

Although we applied a state-of-the-art analytical technique to characterize latent symptom classes, there is some subjectivity to identifying how many classes are sufficient; confidence in the final class solution was based on a combination of statistical indices of fit, the clinical meaningfulness and conceptual interpretability of the class structure [20]. This was a cross-sectional study thus assessing the temporal stability of the identified symptom classes and whether baseline antecedent variables remain predictive of membership in these classes will be important in future studies. Replication of these symptom classes should be confirmed in other larger COPD samples with less restrictive inclusion criteria such as ours where patients were excluded if they reported a known disorder with an underlying inflammatory mechanism. While the high sensitivity assays can measure circulating cytokines at very low levels, the clinical relevance of these very low levels could be questioned. Finally, we did not include the breadth of symptoms that patients with COPD experience; however, the five symptoms we did include captures the most prevalent and distressing symptoms for this population [33].

## Conclusions

Use of a universal patient reported outcome metric such as symptoms to risk-stratify individuals is increasingly important when nearly two thirds of older adult patients have multi-morbidities and disease specific-metrics alone are insufficient to represent the heterogeneity within specific clinical populations [34]. We were able to identify four distinct symptom classes (low physical/psychological, low physical/moderate psychological, high physical/moderate psychological, and high physical/psychological) based on five common symptoms in this sample of patients with stable COPD. Overall, we did not find any consistent association between the 14 serum biomarkers of systemic inflammation with symptom severity. While additional work is needed to test the reliability of these symptom classes, their biological drivers and their validity for prognostication and tailoring therapy in larger samples in longitudinal studies, there is clearly a subgroup of young COPD patients with a long standing depression history who need more intensive and integrative management of both their mental and physical health.

## Abbreviations

AIC: Akaike’s Information Criterion
BIC: Bayesian Information Criterion
CASCADE: COPD Activity: Serotonin Transporter, Cytokines and Depression
COPD: Chronic obstructive pulmonary disease
CRP: High sensitivity C-reactive protein
CRQ: Chronic Respiratory Questionnaire
ECLIPSE: Evaluation of COPD Longitudinally to Identify Predictive Surrogate Endpoints
FEV_1_/FVC: Forced expiratory volume in one second to forced vital capacity ratio
GM-CSF: Granulocyte macrophage-colony stimulating factor
High Psych: High psychological
High-Phys: High physical
IL: Interleukin-1, IL-2, IL-4, IL-5, IL-6, IL-7, IL-8, IL-10, IL12, IL13
INF: Interferon
Low-Phys: Low physical
Low-Psych: Low psychological
Mod Psych: Moderate psychological
NETT: National Emphysema Treatment Trial
SCID: Structured Clinical Interview for Depression
SOBQ: Shortness of Breath Questionnaire
TNF-α: Tumor necrosis factor
